# Supplementary material for: Targeted depletion of uterine glandular Foxa2 induces embryonic diapause in mice
Source: eLife. 2022 Jul 21;11:e78277. doi: 10.7554/eLife.78277 (PMC9355561; doi:10.7554/eLife.78277)
Supplement: Supplementary file 1. — Implantation sites in Foxa2f/f, Foxa2f/fLtfCre+, and Foxa2f/fPgrCre+females on day 8 of pregnancy. [file elife-78277-supp1.docx]

Supplementary file 1

Supplementary table 1. Implantation sites in *Foxa2^f/f^*, *Foxa2^f/f^Ltf^Cre+^* and *Foxa2^f/f^Pgr^Cre+^* females on day 8 of pregnancy.

|  | **No. of mice** | **No. of mice with IS (%)** | **No. of IS** | **No. of mice without IS (%)** | **No. of blastocysts recovered** |
| --- | --- | --- | --- | --- | --- |
| ***Foxa2^f/f^*** | 6 | 6 (100%) | 11.5 ± 0.3 | 0 (0%) | N/A |
| ***Foxa2^f/f^Ltf^Cre/+^*** | 6 | 1 (16.7%) | 8 | 5 (83.3%) * | 5.4 ± 0.7 |
| ***Foxa2^f/f^Pgr^Cre/+^*** | 7 | 3 (42.9%) | 4.6 ± 1.2 | 4 (57.1%) * | 4.0 ± 0.3 |

**P* < 0.05, Fisher’s exact probability test.
